# Improving Accessibility for Core Trainee Doctors to Fulfil Their ECT Competencies (a Mandatory Requirement as Part of the Core Curriculum)

**DOI:** 10.1192/bjo.2026.11276

**Published:** 2026-06-30

**Authors:** Catherine Siju, Amar Hujan, Joanna Pegg

**Affiliations:** Black Country Healthcare NHS Foundation Trust, Dudley, United Kingdom

## Abstract

**Aims:**

Feedback was collected from Core Trainees (CTs) working at Black Country Healthcare Foundation Trust to survey their satisfaction with the way that ECT was being delivered at the time. Primary aim was to assess/rectify CT issues that were causing difficulties in achieving their ECT competencies with secondary aims to improve delivery of ECT from a patient/trust perspective as well.

**Methods:**

Initially a prequestionnaire was delivered to determine the extent of the need and whether CTs would be positive towards a separate rota.

Afterwards we implemented a new specific rota for ECT clinic, over a trial period of 3 months, where a dedicated CT would cover the entire ECT list rather than the rostered on-site on-call doctor (if that doctor was agreeable to this). This was tracked via a live Excel document that was regularly updated to show the ECT clinics that were and weren’t available and CTs were able to put themselves down for available clinics.

We then performed a post-trial survey with comparable questions to assess for benefit of the rota and then asked the cohort for their views on the ECT rota itself and whether it should be reimplemented (as well as advice on improving that process if it were to restart).

**Results:**

Pre-trial responses showed that 65% and 71% of CTs had not received ECT induction or had competencies signed off, respectively. Over half had difficulties achieving competencies and 94% thought a more structured programme would help with achieving these.

Post-trial feedback was overwhelmingly positive with significant improvements in all measured parameters. Particular highlights being how 100% of CTs surveyed had now performed ECT vs 65% previously; and 94% favoured re-introducing the ECT rota. The percentage of CTs that had their ECT competencies signed off more than doubled (from 36% to 67%) and the vast majority felt the ECT rota made things better and less stressful whilst it was running.

**Conclusion:**

Our conclusion was that a separate ECT rota was required going forward, particularly considering the Royal College's current ECT training requirements for CTs. Itwas recommended – and agreed to – that a centralised rota managed through the postgraduate medical rota coordinators would be beneficial. These findings were discussed and presented at the Tutors and Trainees meeting, attended by College Tutors and the Head of School.

